# Novel Binding Mechanisms of Fusion Broad Range Anti-Infective Protein Ricin A Chain Mutant-Pokeweed Antiviral Protein 1 (RTAM-PAP1) against SARS-CoV-2 Key Proteins in Silico

**DOI:** 10.3390/toxins12090602

**Published:** 2020-09-17

**Authors:** Yasser Hassan, Sherry Ogg, Hui Ge

**Affiliations:** 1 Ophiuchus Medicine Inc., Vancouver, BC V6B 0M3, Canada; 2 Biotechnology, Johns Hopkins University, AAP, Baltimore, MD 21218, USA; slojac@gmail.com; 3 AscentGene Inc., Gaithersburg, MD 20878, USA; hge@ascentgene.com

**Keywords:** fusion proteins, ricin, pokeweed antiviral protein, COVID-19, SARS-CoV-2, antiviral agent, ribosome-inactivating proteins

## Abstract

The deadly pandemic named COVID-19, caused by a new coronavirus (SARS-CoV-2), emerged in 2019 and is still spreading globally at a dangerous pace. As of today, there are no proven vaccines, therapies, or even strategies to fight off this virus. Here, we describe the in silico docking results of a novel broad range anti-infective fusion protein RTAM-PAP1 against the various key proteins of SARS-CoV-2 using the latest protein-ligand docking software. RTAM-PAP1 was compared against the SARS-CoV-2 B38 antibody, ricin A chain, a pokeweed antiviral protein from leaves, and the lectin griffithsin using the special CoDockPP COVID-19 version. These experiments revealed novel binding mechanisms of RTAM-PAP1 with a high affinity to numerous SARS-CoV-2 key proteins. RTAM-PAP1 was further characterized in a preliminary toxicity study in mice and was found to be a potential therapeutic candidate. These findings might lead to the discovery of novel SARS-CoV-2 targets and therapeutic protein structures with outstanding functions.

## 1. Introduction

A new global pandemic disease named COVID-19 has emerged and is still spreading at alarming rates at the time of this report. COVID-19 can cause severe symptoms such as damaging inflammatory response, fever, or severe respiratory illness and lead to death. The causative agent of COVID-19 was found to be a novel coronavirus closely related to the severe acute respiratory syndrome coronavirus (SARS-CoV) based on the latest phylogenetic analysis [1,2,3]. There are some major essential differences in their genetic makeup that led to their different behaviors. SARS-CoV-2, as it is called now, appears to have high transmissibility from person to person, and antibodies that could inhibit SARS-CoV are not functional on SARS-CoV-2 [2,4,5]. Despite global efforts, we still lack an effective antiviral strategy, drug, or vaccine to fight this virus, with the growing fear that SARS-COV-2 may become another endemic virus in our communities.

To lower the costs and speed up the drug discovery phase, numerous researchers have used in silico tools such as protein–ligand docking software to screen for traditional compounds that could bind to and inhibit the key proteins present in SARS-CoV-2, highlighting their potential antiviral activity [6]. The major targets for these compounds include SARS-CoV-2 key proteins 3-chymotrypsin-like protease (Mpro), papain-like protease (PLpro), RNA-dependent RNA polymerase (RdRp), small envelope protein (E), membrane protein (M), and spike (S) proteins. The S proteins directly interact with human angiotensin-converting enzyme (ACE2), allowing the virus to enter the cells. The S protein is a class I fusion protein consisting of S1 and S2 domains with the receptor-binding domain (RBD) located on the S1 domain [4]. The RBD is the main target of antibodies and fusion inhibitors in development such as the human convalescent COVID-19 patient-origin B38 antibody (B38) and plant lectin griffithsin (GRFT). Here, we report the in silico potent binding mechanisms against SARS-CoV-2 key proteins of a previously discussed novel broad-spectrum anti-infective fusion protein between a mutant of the ricin A chain and pokeweed antiviral protein isoform 1 (RTAM-PAP1) from seeds of *Ricinus communis* and leaves of *Phytolacca americana*, respectively [7]. RTAM-PAP1 activity was compared with that of the B38, ricin A chain (RTA), pokeweed antiviral protein isolated from leaves (PAP1), and GRFT. Their binding capacities were evaluated against the major key proteins of SARS-CoV-2 using the latest peptide-ligand docking software [8,9,10,11,12,13].

## 2. Results

**Figure 1:**
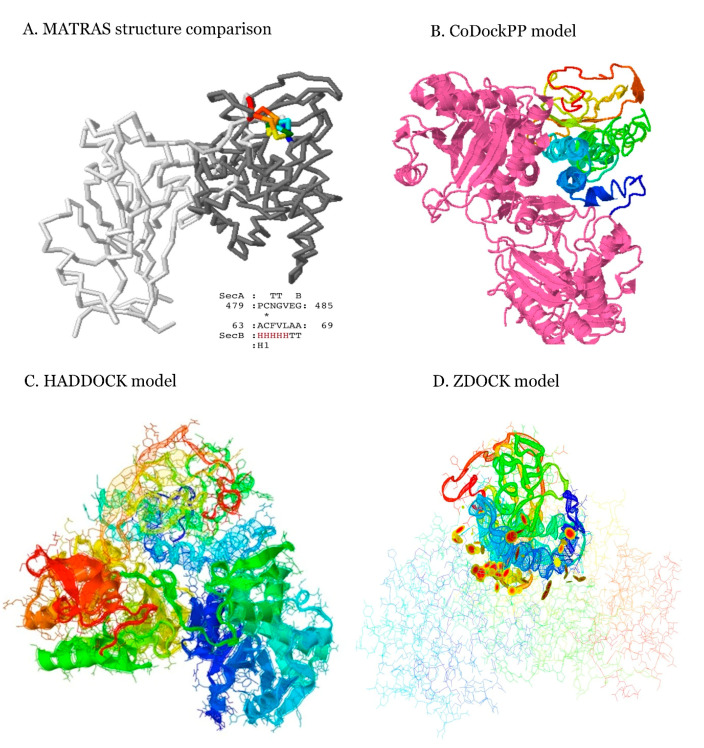
(**A**) Three-dimensional (3D) structure comparison by MATRAS of M protein epitope with S1 protein. M epitope sequence (SecB) is not similar to S1 RBD with only one residue in common (marked with an “*”), yet the structure is similar (depicted in rainbow colors). (**B**) Top model generated by CoDockPP of RTAM-PAP1 (magenta, RTAM being on the upper left side) in complex with M protein (in rainbow colors). (**C**) Top model generated by HADDOCK2.2 with a score of −163.8 (±8.3). PAP1 is on the left side to denote its more important role than in other models and RTAM on the right side (from right to left, N to C terminal in blue to red). M protein is on top and in less solid rainbow colors (N to C terminal in blue to red). (**D**) The top model generated by ZDOCK with RTAM-PAP1 in backbone format (N to C terminal in blue to red). The colored disks depict the binding contact sites. The disks indicate where the van der Waals radii of atoms overlap and the colors how close the contact is: yellow = close, orange = touching, and red = overlapping (models viewed using Jmol).

The three-dimensional (3D) structure of RTAM-PAP1 prediction was obtained as previously described [7], and those of RTA, PAP1, B38, and GRFT were retrieved in protein data bank (PDB) format from the Research Collaboratory for Structural Bioinformatics (RCSB) website (https://www.rcsb.org/). A knowledge-based scoring docking prediction was performed for all the compounds against S, S1 RBD, and M using CoDockPP global docking. An additional run was conducted for ACE2 and human SARS-CoV antibody CR3022 against S1 RBD as a reference [5]. The 3D structures of all the key proteins and ACE2 were already available from the software site in this “COVID-19 targets docking only” version. The peptide/antibody–ligand version was used, as small molecules docking software is not suited for these types of compounds. The generated 3D models of B38, ACE2, and CR3022 bound to S1 RBD were comparable to available crystallography of the same complexes in RCSB (access: 7BZ5, 6M0J, and 6W41, respectively) with respective root mean square deviation (RMSD) varying from 0.7 to 4.311 (A), 0.121 to 2.196 (A), and 0.058 to 3.206 (A). However, the greater binding affinity and fusion inhibiting activity of B38 compared to CR3022 for S1 RBD was observed in accordance with published in vitro results [1,3,5]. B38 was found to have a dissociation constant of 70.1 nM with complete inhibition of ACE2 binding to S1 RBD compared to CR3022′s dissociation constant of 115 nM with no inhibition of ACE2 binding. The difference in inhibition of ACE2 binding to S1 RBD is due to their binding conformation to S1 RBD. However, ACE2 binding to S1 RBD was found to have the smallest dissociation constant in literature, with a value ranging from 4 to 15 nM. The results for the first and last models (out of the top 10 generated) of each compound in complex with S, S1 RBD, and M are presented in Table 1. B38 has the highest overall binding affinity of the lot with a binding energy ranging from −449 to −300 kcal/mol, as expected. ACE2′s binding energy was between −314 to −246 kcal/mol for S1 RBD. RTAM-PAP1 is comparable to B38, with an overall higher binding affinity (lower binding energy) than all of the other compounds tested against the S, S1 RBD, and M key proteins, sometimes higher than B38 with −469 kcal/mol for M, for example. The high binding affinity of RTAM-PAP1 and B38 to S, S1, and M may be explained by the M epitope being very similar in structure to S1 RBD (Figure 1A) [1,3]. RTA binding affinity is similar to RTAM-PAP1 to a certain extent and GRFT and PAP1 are very comparable.

The same higher binding affinity behavior for RTAM-PAP1 was observed with Mpro, PLpro, E, and RdRp when compared to PAP1, GRFT, and RTA (Table 2). All of the tested compounds showed potentially inhibiting binding conformations to the various key proteins based on the 3D structures of the complexes formed (results not shown). These results indicated that the fusion between RTAM and PAP1 allowed RTAM-PAP1 to be more stable across the different possible binding conformations with a higher binding affinity than either of its moieties alone when in complex with SARS-CoV-2 key proteins. 

B38 was found to have a 50% inhibition of the cytopathic effect (EC50) against SARS-CoV-2 simultaneous infection in Vero cells in vitro at the concentration of 0.177 µg/mL. It was further demonstrated that B38 was effective in mice post-infection [1]. GRFT was found to have low pre-infection EC50 on different strains of SARS-CoV in cytoprotection (CPE) assays in vitro (0.6–1.2 µg/mL) and effective in mice pre-infection [14]. RTA was shown in the literature to have a high binding affinity to many viral proteins [15,16]. PAP1 has a broad range of antiviral activity against numerous infections both in vitro and in clinical trials [17,18]. An earlier different version of RTAM-PAP1 was shown to have potent broad range antiviral activity at low post-infection EC50 (0.002–12.3 µg/mL) against human immunodeficiency virus-I (HIV), hepatitis B virus (HBV), hepatitis C virus (HCV), Zika virus (Zika), and human coronavirus 229E (HCoV229E) in CPE assays in vitro [7,19]. RTA and PAP1 produce a drastic increase in viral inhibition activity if administered pre-infection both in vitro and in vivo at sub-toxic dosages [20,21,22,23], with potent antiviral mechanisms, from viral DNA/RNA depurination, viral proteins synthesis inhibition, viral cell entry inhibition, to apoptosis induction of infected cells via a preferential virus-infected cell entry mechanism [7].

This high affinity of RTAM-PAP1 to many key proteins of SARS-CoV-2 is uncommon. Yet, the most surprising part of the generated models was the discovery of unique binding mechanisms of RTAM-PAP1 with potential inhibiting activity by hindering viral entry and cellular machinery. This discovery might explain the previously observed gain of function of RTAM-PAP1 [7] via the acquired ability to simultaneously bind the target with both moieties with high affinity, i.e., increasing the docking sites from 86 to 102 for single moiety binding and simultaneous binding to S1 RBD, for example. To confirm these findings, RTAM-PAP1 was run against SARS-CoV-2 S1 and M using different docking programs (ZDOCK and HADDOCK2.2) with the known active residues in RCSB. The synergetic binding of RTAM-PAP1 was confirmed, and the generated models for M are shown in Figure 1B–D. Although the model generated by HADDOCK2.2 returned a more important role for PAP1 than RTAM, the simultaneous binding of both moieties can clearly be seen when in complex with M, with an increase in docking sites from 62 for single moiety binding to 96 for simultaneous binding of both moieties (ZDOCK model). This might significantly increase RTAM-PAP1′s potential anti-SARS-CoV-2 activity. We concluded from these results and those previously acquired in vitro that the fusion of RTAM and PAP1 via the flexible linker conferred greater structure stability, enhanced activities, new binding sites and mechanisms, and, potentially, novel functions to RTAM-PAP1.

For those reasons, the decision to produce highly purified RTAM-PAP1 protein was taken to conduct a short toxicity study in BALB/c mice to determine the potential maximum tolerated dose.

**Figure 2:**
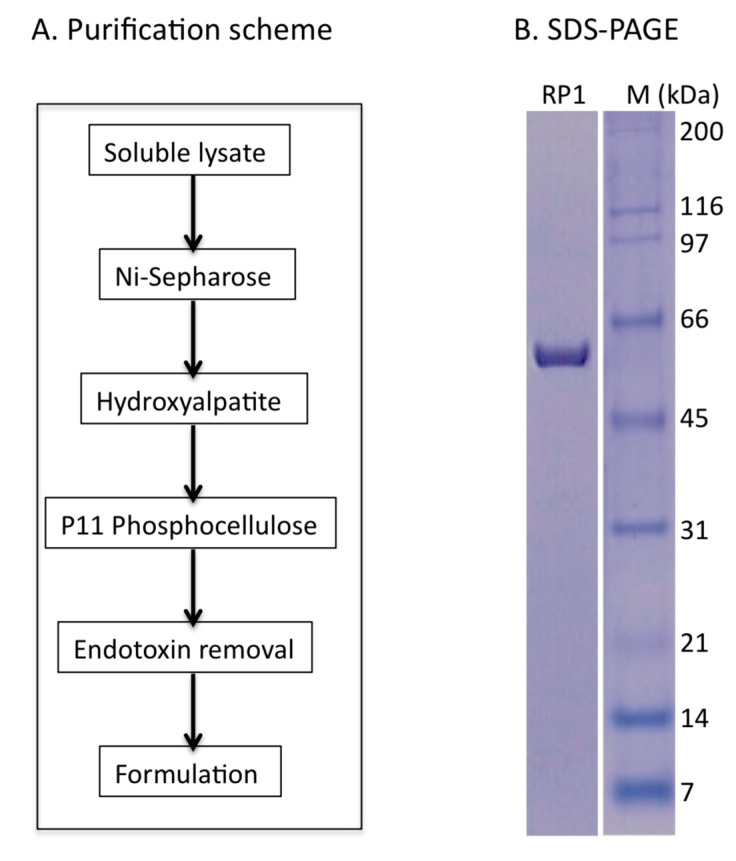
(**A**) Purification scheme and (**B**) purified RTAM-PAP1 protein (RP1). “M” represents protein standards in kilodalton.

The protein production went well and followed a scheme previously used [7] with the addition of an endotoxins removal step after purification, as shown in Figure 2A. Highly purified 6-His tag RTAM-PAP1 was obtained (>95% purity), as shown in Figure 2B. The bioactivity of the proteins was confirmed using a cell-free protein synthesis inhibition assay at three different concentrations in duplicate and yielded a half maximal inhibitory concentration (IC50) of 0.06 nM at 60 min incubation time, in line with previous results [7] confirming the time- and concentration-dependent inhibitory activity of RTAM-PAP1 on protein synthesis (data available upon request).

**Figure 3:**
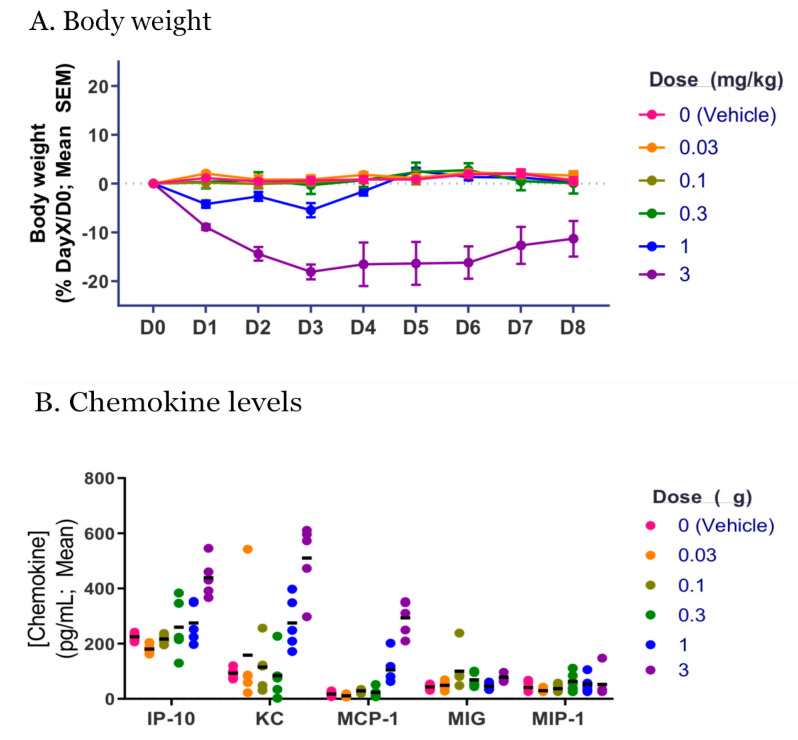
(**A**) Body weight of mice measured daily following administration of various single bolus injection concentrations compared to control. (**B**) Serum chemokine levels measured 3hrs after administration of various single bolus injection concentrations compared to control.

The mice were administered the highly purified RTAM-PAP1 with the 6-His tag and tolerated up to 1 mg/kg with no observable adverse effects. Adverse clinical signs were observed (i.e., weight loss, piloerection, etc.) at a single bolus intravenous administration of 3 mg/kg of RTAM-PAP1 with up-regulation of IP-10, KC, and MCP-1 chemokines from 14 cytokines/chemokines assessed (Figure 3). These results are in line with previously described homopolymers of ribosome-inactivating proteins and confirm an in vivo behavior intermediate between native ribosome-inactivating proteins and immunotoxins [24,25].

## 3. Conclusions

In conclusion, given the very high affinity for SARS-CoV-2 key proteins, the previous antiviral results in vitro, the newly discovered mechanisms, the preliminary in vivo profile, potent bioactivities across the assays, and preferential entry into virus-infected cells as opposed to non-infected cells, we opine that this novel chimeric protein composed by two ribosome-inactivating proteins be tested against SARS-CoV-2 in vitro and in vivo. It would be the first therapeutic testing using this particular strategy against COVID-19 and might make a difference at subtoxic dosages as well as open the doors for the discovery of novel SARS-CoV-2 targets, therapeutic protein structures, and foundations for protein engineering. Those types of fusion proteins are able to outperform immunotoxins with lower production costs and less toxicity.

## 4. Materials and Methods

### 4.1. Protein Modeling

#### 4.1.1. Generation of 3D Structures

The predicted molecular 3D structure of RTAM-PAP1 was already available from previous work [7] and is available in the supplementary files (PDB file S1). The 3D models for RTA, PAP1, B38, CR3022, and GRFT were retrieved from RCSB (https://www.rcsb.org), in PDB format with the following PDB ID: 4MX5, 1PAG, 7BZ5, 6W41, and 3LL2, respectively). The 3D models for S, Mpro, PLpro, ACE2, RdRp, E, and M were retrieved directly from the CoDockPP site (https://ncov.schanglab.org.cn/). The 3D models for the B38-S1, CR3022-S1 and ACE2-S1 complexes were also retrieved from RCSB, 7BZ5, 6M0J, and 6LZG, respectively, for comparison with CoDockPP outputs.

#### 4.1.2. Structure Modeling

The structure of the bound complexes was generated by CoDockPP using the ambiguous peptide-ligand computations [8,9,10]. The B38-S1, CR3022-S1, and ACE2-S1 were compared by superimposition on the available crystallography in RCSB using MATRAS pairwise 3D alignment (http://strcomp.protein.osaka-u.ac.jp/matras/matras_pair.html). Additional models of the RTAM-PAP1-S1 and RTAM-PAP1-M complexes were generated using ZDOCK and HADDOCK2.2 [11,12,13] with the available RCSB active residues as inputs for each protein. The putative active residues for RTAM-PAP1 were previously generated [7] and are available in the supplementary files (Figure S1). All models were viewed using Jmol.

### 4.2. Escherichia coli In Vivo Expression System and Rabbit Reticulate Lysate Protein Synthesis Inhibition

#### 4.2.1. Protein Expression and Purification

RTAM-PAP1 was produced and purified as previously described [7]. The vector pET30a-6H-RPAP1 was generated and validated by DNA sequencing before being transformed into *E. coli* BL21 (DE3) cells (NEB). Expression of the proteins was examined from individual clones and analyzed by either Western blot using a monoclonal antibody specific to ricin A chain (ThermoFisher, RA999, Frederick, MD, USA) or SDS gel stained with Coomassie blue (ThermoFisher, Frederick, MD, USA). Optimal conditions were determined and protein production was induced in the presence of 1 mM isopropyl beta-D-1-thiogalactopyranoside (IPTG) from 1 L culture. The bacteria were then harvested by centrifugation, followed by lysing the cell pellets with 50 mL of lysis buffer (50 mM Tris•Cl, 150 mM NaCl, 0.2% Triton X100, and 0.5 mM ethylenediaminetetraacetic acid (EDTA). After sonication (3 × 2 min), the soluble lysates were recovered by centrifugation at 35,000 rpm for 40 min. The soluble proteins were then purified by the combination of affinity and conventional chromatographic methods from soluble lysates (please contact the authors for more details). The purification of the native RTAM-PAP1 from soluble lysate was achieved by affinity versus His-tag on the Ni-sepharose column (GE Healthcare). After extensive washing with the lysis buffer, loosely bound proteins were eluted with the lysis buffer containing 40 mM Imidazole (I40). RTAM-PAP1 proteins were eluted with the elution buffer (20 mM Tris•Cl, pH7.9, 100 mM NaCl, 1 mM EDTA, and 300 mM Imidazole). A second purification step using the hydroxyapatite column (GE Healthcare, Piscataway, NJ, USA) was used to further separate RTAM-PAP1 from co-purified host proteins. A third purification step, gel filtration on a fast protein liquid chromatography (FPLC) column of Superose 12 (GE Healthcare, Piscataway, NJ, USA), was necessary to completely remove degraded or/and premature protein products [7]. The resulting mixture was subjected to the endotoxin removal process using a proprietary technology developed by AscentGene until the endotoxin level was less than 10 EU/mL. The final product was formulated in the buffer containing 20 mM HEPES-Na (4-(2-hydroxyethyl)-1-piperazineethanesulfonic acid sodium salt), pH 7.9, 200 mM NaCl, 0.2 mM CaCl_2_, and 0.5 mM EDTA.

#### 4.2.2. Rabbit Reticulate Lysate Protein Synthesis Inhibition

The inhibitory activities of RTAM-PAP1 were tested by using the Rabbit Reticulate Lysate TnT^®^ Quick Coupled Transcription/Translation System and the Luciferase Assay System (Promega, Madison, WI, USA). Each transcription/translation reaction was performed according to the instructions for use (IFU) in the presence of a T7 Luciferase reporter DNA, and the luciferase expression level was determined with a Wallac Microplate Reader. Transcription/translation were run at three different concentrations to confirm bioactivity [7].

### 4.3. Preliminary Toxicity Study on Mice

#### 4.3.1. BALB/c Mice

Female BALB/c mice, aged 6–8 weeks (Charles River Laboratories, Saint-Constant, QC, Canada), were used in this study. Female mice were housed in groups of five in individually ventilated cages. The mice were maintained at the National Research Council Canada (NRC) in accordance with the guidelines of the Canadian Council on Animal Care. All procedures performed on animals in this study were in accordance with regulations and guidelines reviewed and approved by the NRC Human Health Therapeutics Ottawa Animal Care Committee.

#### 4.3.2. Animal Procedures

RTAM-PAP1 was administered by intravenous (IV) bolus injection of 0.25 mL into the tail vein in a dose-escalating manner (0.03, 0.1, 0.3, 1, and 3 mg/kg). Control mice received an equivalent volume of vehicle. Dosing was staggered to allow for an initial assessment of tolerability at a particular dose level prior to escalating to a higher dose level. Mice were weighed and evaluated daily for clinical signs for 8 days following administration of RTAM-PAP1. Data were analyzed using GraphPad Prism (GraphPad Software, Inc., San Diego, CA, USA). Statistical significance of the difference between groups was calculated by 1- or 2-factor ANOVA followed by post-hoc analysis. Differences were considered to be not significant at *p* > 0.05.

## 5. Patent

Hassan, Y. and Ogg, S. WO/2019/204902, The World Intellectual Property Organization (WIPO), 2019.

## Figures and Tables

**Table 1:** The binding energies in kcal/mol for the models generated by CoDockPP for each compound in complex with the outer virus envelope proteins. Top 1 is the model with the lowest binding energy (highest binding affinity) and the top 10 is the 10th model with the lowest binding energy. The lowest energy for the top 1 and top 10 models for each complex is in bold.

Key Proteins	S1 RBD		Spike Trimer		Membrane Protein	
	Top 1	Top 10	Top 1	Top 10	Top 1	Top 10
ACE2	−314	−246				
Compounds						
CR3022	−347	−285				
B38 Antibody	**−367**	**−300**	**−385**	−297	−449	−359
GRFT	−273	−239	−283	−250	−280	−265
RTAM-PAP1	−322	−282	−325	**−298**	**−469**	**−393**
RTA	−322	−278	−313	−275	−387	−348
PAP1	−269	−233	−281	−255	−300	−266

**Table 2:** The binding energies in kcal/mol for the models generated by CoDockPP for each compound less the antibody with the viral proteins important for cellular machinery. Top 1 is the model with the lowest binding energy (highest binding affinity) and the top 10 is the 10th model with the lowest binding energy. The lowest energy for the top 1 and top 10 models for each complex is in bold.

Key Proteins	Mpro		Plpro		RdRp		E Protein	
	Top 1	Top 10	Top 1	Top 10	Top 1	Top 10	Top 1	Top 10
Compounds								
GRFT	−228	−198	−234	−209	−267	−248	−258	−242
RTAM-PAP1	**−301**	**−266**	−276	**−259**	**−332**	**−301**	**−363**	**−306**
RTA	−299	−260	**−283**	−254	−304	−277	−314	−281
PAP1	−246	−207	−225	−188	−244	−228	−244	−229

## Supplementary Materials

The following are available online at https://www.mdpi.com/2072-6651/12/9/602/s1, Figure S1: RTAM-PAP1 active sites. PDB file S1: RTAM-PAP1 3D structure.
