# Supplementary material for: Novel Binding Mechanisms of Fusion Broad Range Anti-Infective Protein Ricin A Chain Mutant-Pokeweed Antiviral Protein 1 (RTAM-PAP1) against SARS-CoV-2 Key Proteins in Silico
Source: Toxins (Basel). 2020 Sep 17;12(9):602. doi: 10.3390/toxins12090602 (PMC7551812; doi:10.3390/toxins12090602)
Supplement: Supplementary file 1 [file toxins-12-00602-s001.zip › toxins-930601 supplementary/toxins-930601 supplementary.pdf]

Supplementary Materials: Novel Binding Mechanisms of Fusion Broad Range Anti-Infective Protein Ricin A Chain Mutant-Pokeweed Antiviral Protein 1 (RTAM-PAP1) against SARS-CoV-2 Key Proteins in Silico

Yasser Hassan, Sherry Ogg and Hui Ge

Ligand binding sites

Click to view	Rank	C-score	Cluster size	PDB Hit	Lig Name	Download Complex	Ligand Binding Site Residues
	1	0.59	138	3px9X	JP3	Rep, Mult	80,81,82,122,123,124,173,177,178,181,210
	2	0.05	22	1pagB	FMP	Rep, Mult	360,361,409,410,411,459,467,493
	3	0.04	13	1oqlA	NAG	Rep, Mult	227,230
	4	0.03	7	1j1mA	TRE	Rep, Mult	14,15,16,66,142,143,146,147,196,198
	5	0.02	4	3hioA	C2X	Rep, Mult	76,79,80,81,82,96,97,101,122,124,125,173,177,178,181,212,213,214,259

Download the residue-specific ligand binding probability, which is estimated by SVM.
Download the all possible binding ligands and detailed prediction summary.
Download the templates clustering results.
(a) **C-score** is the confidence score of the prediction. C-score ranges [0-1], where a higher score indicates a more reliable prediction.
(b) **Cluster size** is the total number of templates in a cluster.
(c) **Lig Name** is name of possible binding ligand. Click the name to view its information in the BioLiP database.
(d) **Rep** is a single complex structure with the most representative ligand in the cluster, i.e., the one listed in the **Lig Name** column.
Mult is the complex structures with all potential binding ligands in the cluster.

Enzyme Commission (EC) numbers and active sites

Click to view	Rank	Cscore^{EC}	PDB Hit	TM-score	RMSD[a]	IDEN[a]	Cov	EC Number	Active Site Residues
	1	0.603	1br6A	0.479	1.15	0.978	0.485	3.2.2.22	178,181
	2	0.495	1d6aA	0.475	0.46	1.000	0.476	3.2.2.22	464,467
	3	0.492	2vlcA	0.473	3.22	0.293	0.516	3.2.2.22	178,181
	4	0.490	2qesA	0.471	0.71	0.770	0.474	3.2.2.22	464,467
	5	0.489	3h5kA	0.470	0.89	0.755	0.474	3.2.2.22	464,467

Figure S1. RTAM-PAP1 active sites. RTAM-PAP1 active sites as determined previously by I-TASSER based on the generated 3D model for RTAM-PAP1. The active sites were used to generate the HADDOCK2.2 and ZDOCK models of RTAM-PAP1 in complex with M.
